# Establishment of a prediction model for malignant probability of pulmonary nodules in an adenocarcinoma-predominant cohort

**DOI:** 10.7717/peerj.21353

**Published:** 2026-06-18

**Authors:** Fengru Li, Xiaobo Yu, Kangqiao Xu, Guoqing Zhang

**Affiliations:** Jiading Branch of Shanghai General Hospital, Shanghai Jiao Tong University School of Medicine, Shanghai, China

**Keywords:** Pulmonary nodules, Clinical feathers, Lung adenocarcinoma, Diagnostic value, Prediction model

## Abstract

**Objective:**

This study aimed to identify associated factors for malignancy in pulmonary nodules and establish a risk prediction model to provide a scientific basis for surgical decision-making.

**Methods:**

Patients with pulmonary nodules who underwent surgical resection were retrospectively enrolled for this study. Demographic data, tumor marker levels, computed tomography (CT) features, and pathological results were collected. Univariate and multivariate analyses were performed to identify independent associated factors and construct a predictive model. The model’s performance was evaluated using the receiver operating characteristic (ROC) curve.

**Results:**

A total of 568 patients were included, comprising 146 benign and 422 malignant nodules. The vast majority of malignant nodules were lung adenocarcinomas (*n* = 406). Multivariate analysis showed that female gender (odds ratio (OR) 1.729; 95% confidence interval (CI) [1.114–2.685], *P* = 0.015), lobulation sign (OR: 2.250, 95% CI [1.301–3.890], *P* = 0.004), burr (OR: 2.965, 95% CI [1.740–5.053], *P* < 0.001), the vascular bundle sign (OR: 6.721, 95% CI [1.971–22.922], *P* = 0.002), the nature of nidus mixed ground glass nodules (OR: 2.627 95% CI [1.050–6.572], *P* = 0.039) and the nature of nidus solid pulmonary nodules (OR: 0.169 95% CI [0.102–0.279], *P* < 0.001) were independently associated with malignancy. The area under the ROC curve was 0.81 (*P* < 0.05, 95% CI [0.767–0.849]), with a sensitivity of 91.0% and specificity of 47.3%.

**Conclusion:**

In this adenocarcinoma-predominant cohort, female sex was a significant predictor of malignancy, suggesting that non-smoking women may benefit from increased attention in lung cancer screening strategies. Further validation in diverse populations is warranted.

## Background

A solitary pulmonary nodule is defined as a round or oval lesion with a diameter ≤3 cm, surrounded by lung parenchyma, and without associated atelectasis, hilar enlargement, or pleural effusion (Gould et al., 2013). Pulmonary nodules (PN) are often incidentally detected on chest computed tomography (CT). While most are benign, a minority represent early-stage lung cancer (Anonymous, 2023). Based on density, nodules are classified as solid, pure ground-glass (pGGN), or mixed ground-glass (mGGN) (Patz Jr et al., 2016). Accurate assessment of the pretest probability of malignancy is crucial in managing patients with pulmonary nodules. Early diagnosis and treatment are key to improving patient prognosis, demonstrating a 20% reduction in lung cancer mortality (National Lung Screening Trial Research Team et al., 2011).

Several prediction models have been developed, such as the Mayo model (Herder et al., 2005; Swensen et al., 1997), the Veterans Administration (VA) model (Gould et al., 2007), or the Broke model (McWilliams et al., 2013). For early-stage lung cancer (stage IA, ≤3 cm, non-metastatic), the 5-year survival rate after surgery is as high as 70–80% (Mazzone et al., 2021). Therefore, establishing an accurate and practical prediction model is of great significance for improving lung cancer outcomes. However, consensus on pulmonary nodule management in Asia suggests that guidelines such as those from the American College of Chest Physicians (CHEST) may have limited applicability in Asian populations due to distinct patient characteristics (Bai et al., 2016). It is recommended to adapt such guidelines to better suit Asian clinical practice. This study aimed to develop a predictive model for pulmonary nodules based on Chinese patient data and compare it with existing models to guide clinical decision-making.

## Participants and Methods

### Study design and population

This study was a retrospective investigation. It was approved by Jiading Branch of Shanghai General Hospital Institutional Review Board, Approval No. is Institutional Ethics Quick [2025]016. Written informed consent was obtained from all participants prior to the experiment. The study included all patients with solitary pulmonary nodules admitted to the Jiading Branch of Shanghai General Hospital between January 2021 and January 2024. The criteria were as follows: (1) This investigation recruited patients presenting with pulmonary nodules sized between 5 and 30 mm, for whom a histopathologic determination of benign or malignant status was made. All diagnoses of malignant and benign nodules were based on the pathological examination of lung tissues acquired through thoracic surgery. (2) Only patients with comprehensive clinical data, laboratory examination **d**ata, and routine lung HRCT scans prior to the surgical procedure were included. (3) Pulmonary nodules were characterized as solitary, round or oval opacities that were entirely encompassed by lung parenchyma and had a diameter of less than three cm, with no enlarged lymph nodes, atelectasis, or pneumonia (McWilliams et al., 2013).

The exclusion criteria were as follows: (1) Patients with a treatment history of pulmonary nodules before surgery. (2) The nodules presented as multiple pulmonary nodules; (2) pleural effusion, atelectasis, or lymph node enlargement was detected; or (3) the pathological diagnosis was ambiguous or it was a metastatic tumour.

### CT scan and diagnostic

Clinical data, including gender, age, smoking history, and cancer history, were gathered. The levels of four tumor markers (CEA, NSE, CYFRA 21-1, and ProGRP) were determined. In CT imaging, GGN lesion can be categorized into pure GGN (pGGN), lesion and mixed GGN (mGGN), lesion. Two experienced radiologists each read the film to observe and analyze the diameter, density, location, and morphological features (including lobulation sign, burr sign, pleural traction sign, vascular bundle sign, nature of nidus) of pulmonary nodules. If there was a difference in the results of the diagnosis between the two doctors, the final diagnosis was obtained after discussion within the department.

### Surgical methods and pathological diagnosis

All patients underwent thoracoscopic surgery (lobectomy or sublobectomy). Pathological diagnosis followed the 2021 lung adenocarcinoma classification (Nicholson et al., 2022), including atypical adenomatous hyperplasia (AAH), adenocarcinoma *in situ* (AIS), minimally invasive adenocarcinoma (MIA), and invasive adenocarcinoma (IAC). AAH and AIS were considered pre-invasive, while MIA and IAC were classified as invasive. Other malignant types included squamous cell carcinoma, adenosquamous carcinoma, and small cell carcinoma.

### Statistical analysis

Statistical analyses were performed using SPSS25.0 and Python 3.11.9. *P*-value < 0.05 was considered significant. Continuous variables were tested for normality using the Shapiro–Wilk test. Normally distributed continuous variables were compared using the independent *t*-test and presented as mean ± SD; non-normally distributed variables were compared using the Mann–Whitney U test and presented as median (interquartile range). Categorical variables were analyzed using the chi-square test or Fisher’s exact test (when expected cell counts were <5). Descriptive statistics were generated and presented as median (interquartile range) or frequency (percentage). All categorical variables were recoded as binary indicators (0 for absence, 1 for presence) prior to analysis. The nodule characteristics (pGGN, mGGN, solid) are a three-categorical variable. Using pGGN as the reference group, two dummy variables were generated: nature mGGN (mGGN = 1, others = 0) and nature solid (solid = 1, others = 0). Missing values were addressed using multiple imputation methods. Single factor was used to analyze the relationship between each factor and the benign and malignant pulmonary nodules. Variables with a *P*-value < 0.05 were selected for multivariate logistic regression analysis. Multivariate logistic regression was used to identify independent associated factors. The receiver operating characteristic (ROC) curve was plotted to evaluate model performance.

## Results

### Clinical characteristics of patients, and comparisons between benign cohort and malignant cohort

Table 1 shows the characteristics of 568 patients with solid pulmonary nodules, of whom 146 (25.7%) had benign nodules and 422 (74.3%) had malignant nodules. The mean age of benign group was 57.97 ± 11.01, the malignant group is 57.75 ± 11.44. More malignant nodule-bearing individuals were female than male (*n* = 274, 64.9%), The location and history of tumors show no statistically significant. The radiological features of the lobulation burr, vascular bundle sign, and mGGN show more likely to be malignance, SPN show more likely to be genign. Age, maximum diameter, recent or history of tumors, smoking history, SCC, Cyfra21-1, NSE, ProGRP and pleural traction sign demonstrated no statistical significance in differentiating between benign and malignant solitary pulmonary nodules.

**Table 1:** Significance test of solitary pulmonary nodule clinical features, imaging features and serum tumor markers.

**Index (Total *n* = 568)**	**Benign (*n* = 146)**	**Malignant (*n* = 422)**	**t/*χ*^2^**	***P*-value**
**Age, y, Mean ± SD**	57.97 ± 11.01	57.75 ± 11.44	*t* = 0.202	*0*.*604*
**Maximum diameter, mm**	11.75 ± 6.12	12.71 ± 6.43	*t* = − 1.638	0.541
**Gender**			*χ*^2^ = 13.100	<0.001
Male (0)	76 (52.1%)	148 (35.1%)		
Female (1)	70 (47.9%)	274 (64.9%)		
**History of cancer**			*χ*^2^ = 3.446	0.063
No (0)	138 (94.5%)	377 (89.3%)		
Yes (1)	8 (5.5%)	45 (10.7%)		
**Cigarette smoker**			*χ*^2^ = 1.356	0.244
No (0)	130 (89%)	389 (92.2%)		
Yes (1)	16 (11%)	33 ( 7.8%)		
**SCC**				0.423
Negative (0)	140 (95.9%)	410 (97.2%)		
High (1)	6 (4.1% )	12 (2.8%)		
**CYFRA 21-1**			*χ*^2^ = 0.004	0.947
Negative (0)	119 (81.5%)	345 (81.8%)		
High (1)	27 (18.5%)	77 (18.2%)		
**NSE**			*χ*^2^ = 0.017	0.898
Negative (0)	118 (80.8%)	339 (80.3%)		
High (1)	28 (19.2%)	83 (19.7%)		
**ProGRP**			*χ*^2^ = 0.04	0.842
Negative(0)	140 (95.9%)	403 (95.5%)		
High (1)	6 (4.1%)	19 (4.5%)		
**Upper lobe**			*χ*^2^ = 3.853	0.05
No (0)	71 (48.6)	166 (39.3%)		
Yes (1)	75 (51.4%)	256 (60.7%)		
**Lobulation sign**			*χ*^2^ = 4.168	0.041
No (0)	118 (80.8%)	305 (72.3%)		
Yes (1)	28 (19.2%)	117 (27.7%)		
**Burr**			*χ*^2^ = 7.421	0.006
No (0)	116 (79.5%)	285 (67.5%)		
Yes (1)	30 (20.5%)	137 (32.5%)		
**Pleural traction sign**			*χ*^2^ = 2.018	0.166
No (0)	120 (82.2%)	323 (76.5%)		
Yes (1)	26 (17.8%)	99 (23.5%)		
**Vascular bundle sign**			*χ*^2^ = 20.020	<0.001
No (0)	143 (97.9%)	353 (73.6%)		
Yes (1)	3 (2.1%)	69 (16.4%)		
**Nature of nidus**			*χ*^2^ = 84.507	<0.001
pGGN (1)	37 (25.3%)	183 (43.4%)		
mGGN (2)	6 (4.1%)	117 (27.7%)		
SPN (3)	103 (70.5%)	122 (28.9%)		

**Notes.**

SSC squamous cell carcinoma antigen pGGN pure ground glass mGGN mixed ground glass nodules SPN Solid pulmonary nodules

Lung adenocarcinoma accounted for the overwhelming majority of the malignant nodules (406/422), of which 22 were classified as AAH, 53 as AIS, 153 as MIA, and 178 as IAC. In addition, there were also eight cases of squamous cell carcinoma, one case of adenosquamous cell carcinoma, four cases of small cell carcinoma, one case of large cell carcinoma, one case of non-small cell lung cancer, and one case of malignant melanoma (Table 2).

**Table 2:** Pathological subtype of malignant pulmonary nodules.

**Subtype**	***n* = 422**
**Lung adenocarcinoma**	406 (96.2%)
AAH	22
AIS	53
MIA	153
IAC	178
**Squamous cell carcinoma**	8
**Adenosquamous cell carcinoma**	1
**Small cell carcinoma**	4
**Large cell carcinoma**	1
**Non-small cell lung cancer**	1
**Malignant melanoma**	1

### Multiple logistic regression analysis of potential and significant predictors of malignancy

When constructing the multivariate logistic regression model, the nature of nidus was a three-category mutually exclusive variable (pGGN, mGGN, and SPN), which was encoded into three dummy variables *via* one-hot encoding: nature of nidus pGGN_1, mGGN_2, and SPN_3. In this study, pGGN was selected as the reference category, as it typically represents the subtype with the lowest risk of malignancy in clinical practice. Using this as a baseline allows for an intuitive interpretation of the changes in malignancy risk for the other subtypes (mGGN and SPN) relative to pGGN. After removing this variable, the VIF for all independent variables was below 1.8, indicating no significant multicollinearity in the model and ensuring reliable regression results. We utilized multivariate logistic regression analysis to evaluate the degree of the influence of various factors on benign and malignant pulmonary nodules, and discovered that the gender, lobulation sign, burr, vascular bundle sign and nature of nidus mixed ground glass nodules, solid pulmonary nodules had statistical significance (*p* < 0.05). These factors were assigned in Table 3.

**Table 3:** Logistic multivariate regression analysis and assignment.

**Variable**	**Assignments**	**VIF**
Gender	male = 0; female = 1	1.5803
Lobulation sign	no = 0; yes = 1	1.5240
Burr	no = 0; yes = 1	1.5136
Vascular bundle sign	no = 0; yes = 1	1.1321
Nature of nidus mGGN	2	1.5427
Nature of nidu SPN	3	1.7108

**Notes.**

VIF variance inflation factor

All VIF values <5 indicate no significant multicollinearity. pGGN was used as the reference category for nature of nidus.

All other potential predictors showed no association with malignancy, and none of them were encompassed in the final model. The female sex was associated with a higher odds of malignancy than the males who have malignant nodules (odds ratio (OR) 1.729; 95% confidence interval (CI) [1.114–2.685], *P* = 0.015). The result also showed that lobulation sign (OR: 2.250, 95% CI [1.301–3.890], *P* = 0.004), Burr (OR: 2.965, 95% CI [1.740–5.053], *P* < 0.001), the vascular bundle sign (OR: 6.721, 95% CI [1.971–22.922], *P* = 0.002), the nature of nidus mixed ground glass nodules (OR: 2.627 95% CI [1.050–6.572], *P* = 0.039) and the nature of nidus Solid pulmonary nodules (OR: 0.169 95% CI [0.102–0.279], *P* < 0.001) (Table 4).

**Table 4:** Logistic multivariate regression analysis of predictors of malignant PN.

**Variable**	***β***	**SE**	**Wald *χ*^2^**	***P*值**	**OR (95% CI)**
Intercept	0.911	0.233	15.24	¡0.001	2.486 (1.574–3.927)
Gender	0.548	0.224	5.96	0.015	1.729 (1.114–2.685)
Lobulation sign	0.811	0.279	8.42	0.004	2.250 (1.301–3.890)
Burr	1.087	0.272	15.97	¡0.001	2.965 (1.740–5.053)
Vascular bundle sign	1.905	0.626	9.27	0.002	6.721 (1.971–22.922)
Nature of nidus mGGN	0.966	0.468	4.27	0.039	2.627 (1.050–6.572)
Nature of nidu SPN	−1.781	0.257	47.86	¡0.001	0.169 (0.102–0.279)

### Establishment of prediction equation model

The clinical prediction model for malignancy in solitary pulmonary nodules represents the probability of malignancy as a function of one clinical and five radiological variables in the following manner:

\begin{eqnarray*}\text{Probability of malignancy}={e}^{\mathrm{x}}/(1+{e}^{\mathrm{x}}) \end{eqnarray*}

\begin{eqnarray*}x=0.911+0.548\times \mathrm{Gender}+0.811\times \text{Lobulation sign}+1.087\times \mathrm{Burr}+1.905\nonumber\\\displaystyle  \times \text{Vascular bundle sign}+0.966\times \mathrm{mGGN}-1.781~\times ~\mathrm{SPN} \end{eqnarray*}

where e is the base of natural logarithms, if the patient is a female gender = 1; if the patient is a male gender = 0, if the pulmonary nodule has Lobulation sign = 1 (otherwise = 0), if the pulmonary nodule has a burr = 1 (otherwise = 0), if CT shows vascular bundle sign of the pulmonary nodule = 1 (otherwise = 0), and if the pulmonary nodule is mGGN = 1 (otherwise = 0), SPN = 1 (otherwise = 0). To facilitate clinical interpretation, we calculated predicted probabilities for representative patient profiles based on the final model (Table 5). The predicted probability of malignancy ranged from 18.7% for low-risk profiles (*e.g.*, male with solid nodule and no suspicious features) to 92.3% for high-risk profiles (*e.g.*, female with mGGN exhibiting multiple malignant signs).

**Table 5:** Clinically meaningful patient profiles with predicted probabilities.

**Profile**	**Gender**	**Nodule type**	**Lobulation**	**Burr**	**Vascular bundle**	**Predicted probability**
1 (High risk)	Female	mGGN	Yes	Yes	Yes	92.3%
2 (Moderate risk)	Female	mGGN	Yes	No	No	65.8%
3 (Low risk)	Male	SPN	No	No	No	18.7%

**Notes.**

Predicted probabilities were calculated using the final logistic regression model: P = e^x^/(1+e^x^), where *x* = 0.911 + 0.548 ×Gender + 0.811 ×Lobulation + 1.087 ×Burr + 1.905 ×Vascular bundle + 0.966 ×mGGN - 1.781 ×SPN.

**Figure 1:**
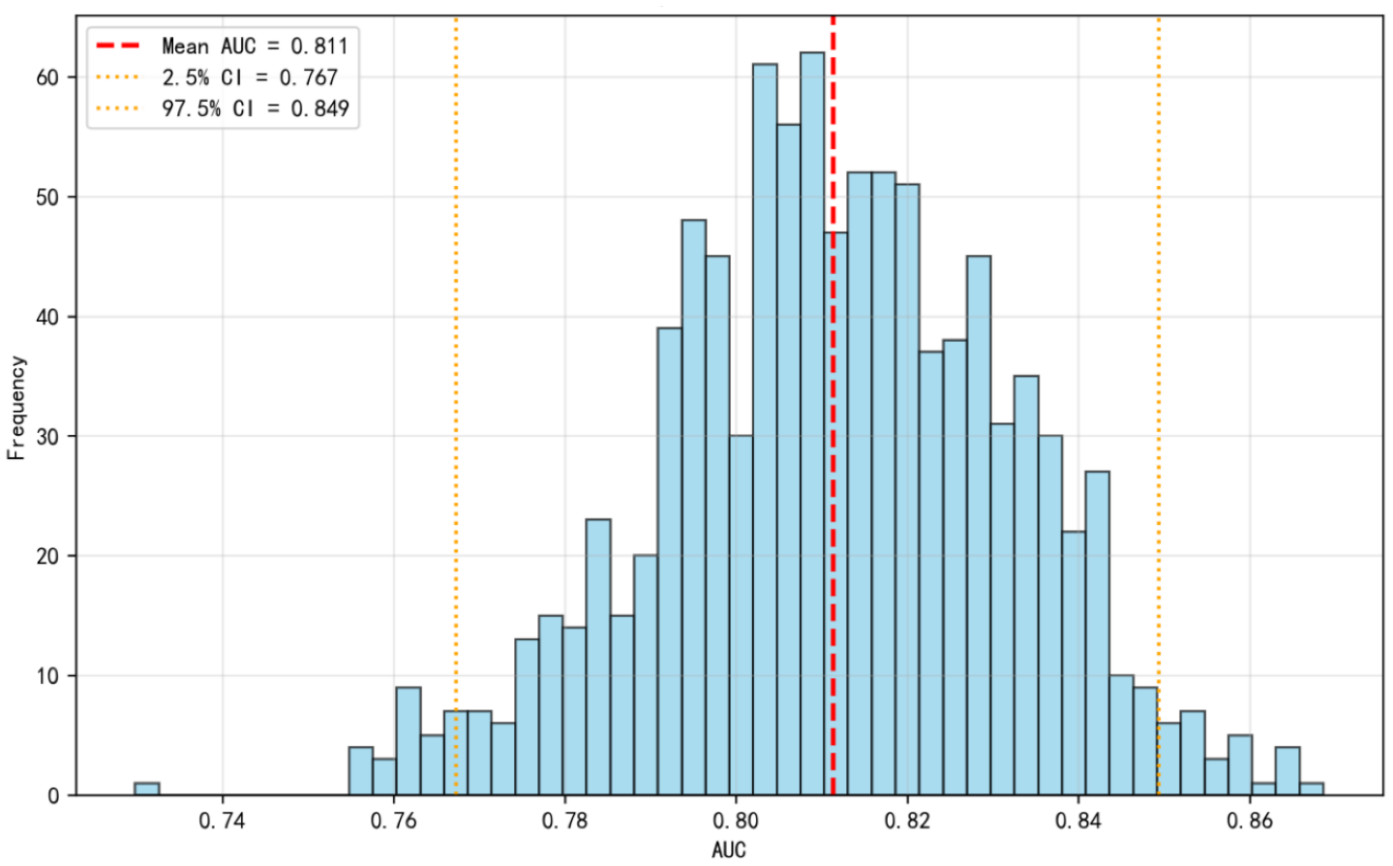
Bootstrap area under the curve (AUC) distribution.

**Figure 2:**
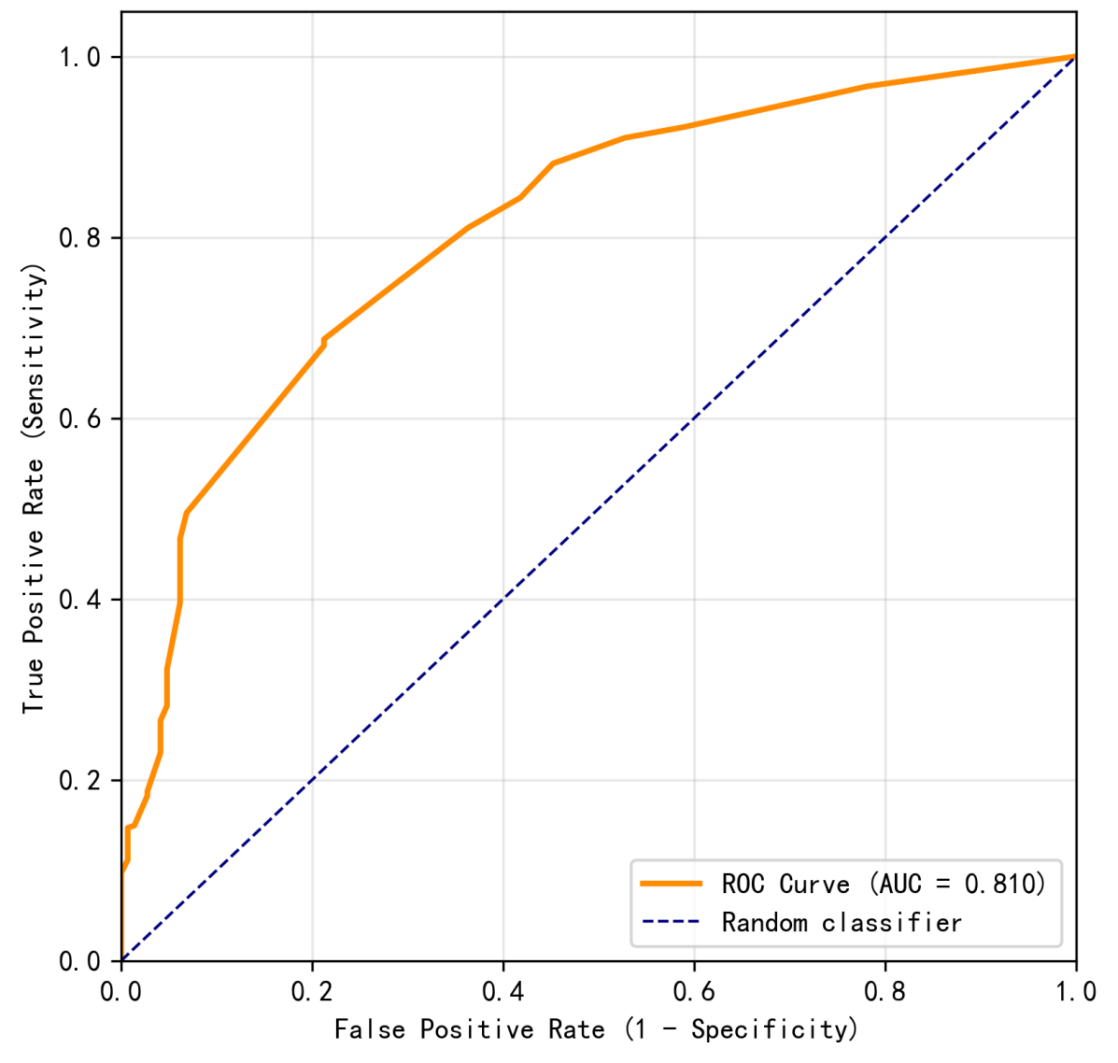
ROC curve.

**Figure 3:**
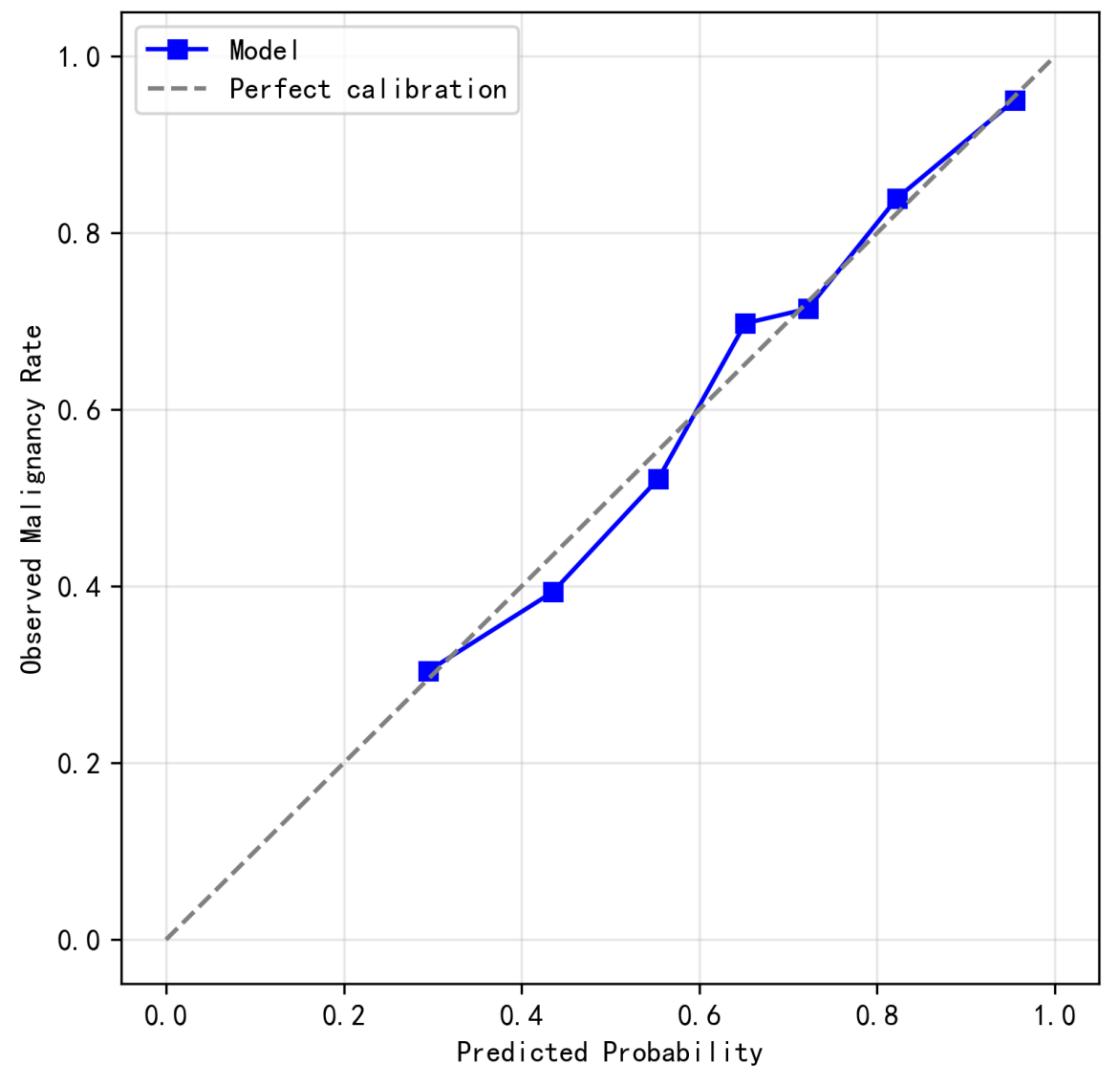
Calibration plot.

**Figure 4:**
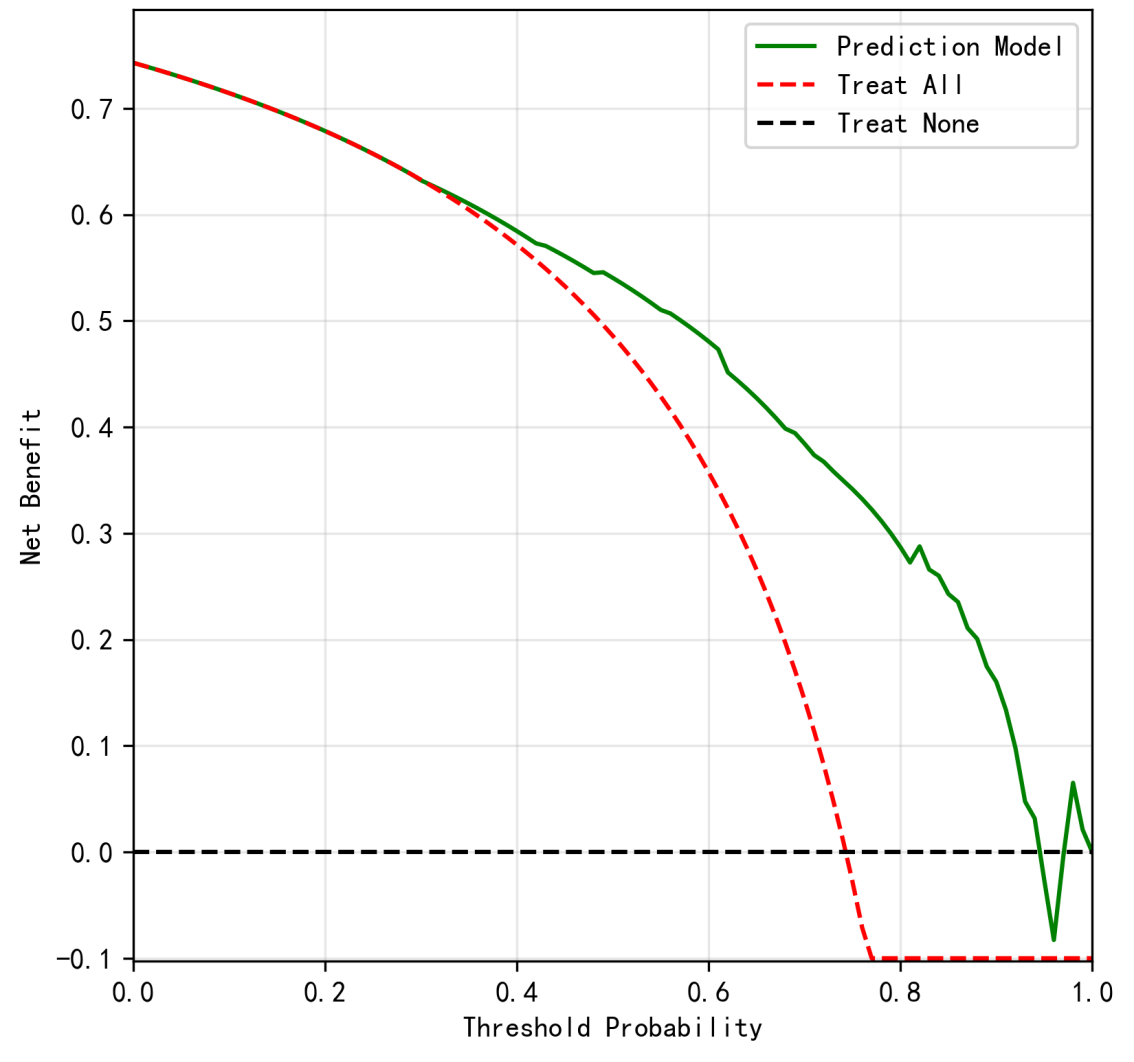
Decision curve analysis (DCA).

Figure 1 is Bootstrap area under the curve (AUC) distribution plot (1,000 Bootstrap resamples). The area beneath the assessed receiver operating characteristic curve amounted to 0.81 (*P* < 0.05, 95% CI [0.767–0.849]). At the juncture, the sensitivity and specificity of the model were respectively 91% and 47.3% (Fig. 2). Figure 3 is calibration plot. Figure 4 is decision curve analysis, DCA.

## Discussion

This study developed a predictive model for distinguishing benign and malignant pulmonary nodules based on Chinese patient data. Our findings highlight the importance of regional characteristics in nodule evaluation. Multivariate analysis identified key imaging and clinical factors, and the model demonstrated good discriminative ability (AUC = 0.81). These findings support a hypothesis that building specific predictive models based on the characteristics of cases in Asia can improve the diagnostic accuracy of solitary pulmonary nodule potentially leading to better patient outcomes (Bai et al., 2016; Adams et al., 2023).

Female sex was identified as a significant predictor of malignancy in our cohort. This association likely reflects the predominance of lung adenocarcinoma in our study population, which is consistent with the higher incidence of this subtype among non-smoking women (Anonymous, 2020). Previous studies have suggested that ground-glass opacities often represent indolent adenocarcinoma, with malignancy probability increasing with solid components (Zhang, Fu & Chen, 2020).

According to current guidelines (Adams et al., 2023), non-smokers are not recommended for lung cancer screening. Although smoking is a well-established associated factor for lung cancer (Moolgavkar et al., 2012), it was not significant in our model, though this finding warrants careful interpretation in context. In this study, the predominance of lung adenocarcinoma in the malignant cases (406 out of 422) and the very low proportion of smoking-related subtypes such as small cell lung carcinoma introduced an imbalance in histological distribution, which may limit the generalizability of the conclusion. The observed sex association likely reflects the higher incidence of lung adenocarcinoma among non-smoking women, rather than representing a universal associated factor across all lung cancer subtypes. Therefore, extrapolation of this conclusion requires validation in future multicenter studies with more balanced and diverse representation of histological subtypes (Zhang et al., 2021), However, this finding also highlights the need to pay closer attention to the risk of lung cancer among non-smoking populations, particularly women, which aligns with the growing scholarly focus on lung cancer in never-smokers.

The developed model incorporates both clinical and radiological variables, providing a practical tool for clinicians (Qiu et al., 2016). But the limitations of this study primarily stem from its retrospective, single-center design. The study required all nodules to be surgically resected with pathological confirmation, enriching for high-risk lesions and excluding those managed by surveillance. This selection bias limits generalizability to broader screening or incidental populations and contributes to a high malignancy prevalence, which influences predictive values. This “enrichment” of cases may lead to seemingly better performance in certain metrics (*e.g.*, positive predictive value), but it simultaneously limits the model’s generalizability for direct application to broader screening populations characterized by a lower pre-test probability of malignancy. While the model’s discriminative ability (AUC) and calibration reported here are relatively robust metrics, external validation in more representative populations is necessary before clinical implementation to further assess its calibration performance and refine prediction thresholds. Future research directions include validation in prospective and multicenter cohorts of incidentally detected nodules, External validation in lower-prevalence cohorts, as well as exploring the integration of the model into clinical decision support systems to evaluate its real-world impact on clinical decision-making and patient outcomes.

## Conclusions

In summary, the integration of clinical features, tumor markers, and high-resolution CT findings improves the discrimination between benign and malignant pulmonary nodules. Our findings highlight the necessity of early cancer screening for non-smoking female populations. More in-depth multicenter studies and research on the generalizability of broad screening populations will continue to refine such models, which are expected to support clinical decision-making and improve patient outcomes.

##  Supplemental Information

10.7717/peerj.21353/supp-1 Supplemental Information 1 Codebook

10.7717/peerj.21353/supp-2 Supplemental Information 2 Raw data

## Additional Information and Declarations

### Data Availability

The following information was supplied regarding data availability: The raw measurements are available in Supplemental Files 1 and 2.

## References

[ref-1] Adams SJ, Stone E, Baldwin DR, Vliegenthart R, Lee P, Fintelmann FJ (2023). Lung cancer screening. Lancet.

[ref-2] (2020). The genomic landscape of nonsmall cell lung carcinoma in never smokers. International Journal of Cancer.

[ref-3] (2023). Evaluation of lung cancer risk among persons undergoing screening or guideline-concordant monitoring of lung nodules in the Mississippi Delta. JAMA Network Open.

[ref-4] Bai C, Choi C-M, Chu CM, Anantham D, Ho JC-M, Khan AZ, Lee J-M, Li SY, Saenghirunvattana S, Yim A (2016). Evaluation of pulmonary nodules: clinical practice consensus guidelines for Asia. Chest.

[ref-5] Gould MK, Ananth L, Barnett PG, Veterans Affairs SNAP Cooperative Study Group (2007). A clinical model to estimate the pretest probability of lung cancer in patients with solitary pulmonary nodules. Chest.

[ref-6] Gould MK, Donington J, Lynch WR, Mazzone PJ, Midthun DE, Naidich DP, Wiener RS (2013). Evaluation of individuals with pulmonary nodules: when is it lung cancer? Diagnosis and management of lung cancer, 3rd ed: American College of Chest Physicians evidence-based clinical practice guidelines. Chest.

[ref-7] Herder GJ, Van Tinteren H, Golding RP, Kostense PJ, Comans EF, Smit EF, Hoekstra OS (2005). Clinical prediction model to characterize pulmonary nodules: validation and added value of 18F-fluorodeoxyglucose positron emission tomography. Chest.

[ref-8] Mazzone PJ, Silvestri GA, Souter LH, Caverly TJ, Kanne JP, Katki HA, Wiener RS, Detterbeck FC (2021). Executive summary: Screening for lung cancer: CHEST guideline and expert panel report. Chest.

[ref-9] McWilliams A, Tammemagi MC, Mayo JR, Roberts H, Liu G, Soghrati K, Yasufuku K, Martel S, Laberge F, Gingras M, Atkar-Khattra S, Berg CD, Evans K, Finley R, Yee J, English J, Nasute P, Goffin J, Puksa S, Stewart L, Tsai S, Johnston MR, Manos D, Nicholas G, Goss GD, Seely JM, Amjadi K, Tremblay A, Burrowes P, MacEachern P, Bhatia R, Tsao MS, Lam S (2013). Probability of cancer in pulmonary nodules detected on first screening CT. New England Journal of Medicine.

[ref-10] Moolgavkar SH, Holford TR, Levy DT, Kong CY, Foy M, Clarke L, Jeon J, Hazelton WD, Meza R, Schultz F, McCarthy W, Boer R, Gorlova O, Gazelle GS, Kimmel M, McMahon PM, De Koning HJ, Feuer EJ (2012). Impact of reduced tobacco smoking on lung cancer mortality in the United States during 1975–2000. Journal of the National Cancer Institute.

[ref-11] Aberle DR, Adams AM, Berg CD, Black WC, Clapp JD, Fagerstrom RM, Gareen IF, Gatsonis C, Marcus PM, Sicks JD, National Lung Screening Trial Research Team (2011). Reduced lung-cancer mortality with low-dose computed tomographic screening. New England Journal of Medicine.

[ref-12] Nicholson AG, Tsao MS, Beasley MB, Borczuk AC, Brambilla E, Cooper WA, Dacic S, Jain D, Kerr KM, Lantuejoul S, Noguchi M, Papotti M, Rekhtman N, Scagliotti G, Van Schil P, Sholl L, Yatabe Y, Yoshida A, Travis WD (2022). The 2021 WHO classification of lung tumors: impact of advances since 2015. Journal of Thoracic Oncology.

[ref-13] Patz Jr EF, Greco E, Gatsonis C, Pinsky P, Kramer BS, Aberle DR (2016). Lung cancer incidence and mortality in National Lung Screening Trial participants who underwent low-dose CT prevalence screening: a retrospective cohort analysis of a randomised, multicentre, diagnostic screening trial. The Lancet Oncology.

[ref-14] Qiu ZX, Cheng Y, Liu D, Wang WY, Wu X, Wu WL, Li WM (2016). Clinical, pathological, and radiological characteristics of solitary ground-glass opacity lung nodules on high-resolution computed tomography. Therapeutics and Clinical Risk Management.

[ref-15] Swensen SJ, Silverstein MD, Ilstrup DM, Schleck CD, Edell ES (1997). The probability of malignancy in solitary pulmonary nodules. Application to small radiologically indeterminate nodules. Archives of Internal Medicine.

[ref-16] Zhang Y, Fu F, Chen H (2020). Management of ground-glass opacities in the lung cancer spectrum. Annals of Thoracic Surgery.

[ref-17] Zhang T, Joubert P, Ansari-Pour N, Zhao W, Hoang PH, Lokanga R, Moye AL, Rosenbaum J, Gonzalez-Perez A, Martínez-Jiménez F, Castro A, Muscarella LA, Hofman P, Consonni D, Pesatori AC, Kebede M, Li M, Gould Rothberg BE, Peneva I, Schabath MB, Poeta ML, Costantini M, Hirsch D, Heselmeyer-Haddad K, Hutchinson A, Olanich M, Lawrence SM, Lenz P, Duggan M, Bhawsar PMS, Sang J, Kim J, Mendoza L, Saini N, Klimczak LJ, Islam SMA, Otlu B, Khandekar A, Cole N, Stewart DR, Choi J, Brown KM, Caporaso NE, Wilson SH, Pommier Y, Lan Q, Rothman N, Almeida JS, Carter H, Ried T, Kim CF, Lopez-Bigas N, Garcia-Closas M, Shi J, Bossé Y, Zhu B, Gordenin DA, Alexandrov LB, Chanock SJ, Wedge DC, Landi MT (2021). Genomic and evolutionary classification of lung cancer in never smokers. Nature Genetics.

