# Supplementary material for: Establishment of a prediction model for malignant probability of pulmonary nodules in an adenocarcinoma-predominant cohort
Source: PeerJ. 2026 Jun 18;14:e21353. doi: 10.7717/peerj.21353 (PMC13283365; doi:10.7717/peerj.21353)
Supplement: Supplemental Information 1 [file peerj-14-21353-s001.docx]

| Factors | Assignments |
| --- | --- |
| Gender | Male 0；Female 1 |
| Pathological results | Benign 0; malignant 1 |
| History of tumors | No 0；Yes 1 |
| Smoking history | No 0；Yes 1 |
| SCC | Normal 0；Above normal level 1 |
| NSE | Normal 0；Above normal level 1 |
| SYFRA21-1 | Normal 0；Above normal level 1 |
| ProGRP | Normal 0；Above normal level 1 |
| upper lobe | No 0；Yes 1 |
| lobulation sign | No 0；Yes 1 |
| Burr | No 0；Yes 1 |
| Pleural traction | No 0；Yes 1 |
| vascular bundle sign | No 0；Yes 1 |
| Calcification sign | No 0；Yes 1 |
| nature of nidus | pGGN, pure ground glass 1; mGGN, mixed ground glass nodules 2; SPN, Solid pulmonary nodules 3 |
